# Disseminated Methicillin-Sensitive Staphylococcus aureus (MSSA) Infection After Acupuncture Treatment

**DOI:** 10.7759/cureus.111305

**Published:** 2026-06-22

**Authors:** Sally Trinh, Janette Zara

**Affiliations:** 1 Internal Medicine, Olive View-University of California, Los Angeles Medical Center (UCLA) Medical Center, Los Angeles, USA; 2 Internal Medicine, University of California, Los Angeles Medical Center (UCLA) Medical Center, Los Angeles, USA

**Keywords:** abscess, acupuncture, alternative medicine, bacaterial infection, bacteremia, complementary medicine, pain relief, sepsis, source control, staphylococcus aureus

## Abstract

Methicillin-sensitive* Staphylococcus aureus *(MSSA) is a common bacterium found on the skin and may cause severe infections, including bacteremia, endocarditis, and osteomyelitis. Studies have shown that despite antibiotic use, 30-day mortality of MSSA bacteremia remains high. Acupuncture involves the insertion of small needles at particular points in the body and is sometimes used to address pain. Acupuncture may be a rare source of MSSA bacteremia with the introduction of bacteria through compromised skin integrity. Here, we present a case of a 54-year-old female patient found to have disseminated MSSA infection, with bacteremia, multiple abscesses, and osteomyelitis near areas of acupuncture in her right hip. Despite antibiotics, the patient did not improve until adequate source control was obtained through drainage of her multiple abscesses and laminectomy for her lumbar spine osteomyelitis from possible hematogenous spread. Even though acupuncture can be a useful modality to treat pain, this case highlights the need for proper sterilization techniques and expert handling to mitigate the risk of infection.

## Introduction

Methicillin-sensitive *Staphylococcus aureus* (MSSA) is a bacterium commonly found on the skin [1]. It can cause localized skin and soft tissue infection and, in some cases, may lead to bacteremia if it enters the bloodstream. MSSA is a predominant cause of bacteremia cases, with studies showing between 25 and 35 cases per 100,000 persons each year [2]. Bacteremia could lead to further complications, including endocarditis and osteomyelitis [3,4]. Studies have shown that *Staphylococcus aureus *is the culprit bacteria for over 30% of endocarditis cases and more than 25% of osteomyelitis cases [3,4]. Despite a wide range of antibiotics to which MSSA is susceptible, including beta-lactams, the 30-day mortality of MSSA bacteremia remains high, ranging from 15% to 30% [5], making it a serious infection. MSSA on the skin can enter the bloodstream through traumatic or iatrogenic compromises in skin integrity.

Traditional acupuncture has roots grounded in the philosophical principles related to Confucianism and Taoism. It involves the insertion of small needles at particular points in the body that help to align “qi” within the body. Qi is meant to be the vital energy that runs through our bodies and encompasses the yin and yang dichotomy that flows through our world [6]. Acupuncture is used to relieve afflictions including chronic low back pain, musculoskeletal pain, and nausea or vomiting [6]. An estimated 2.2% of people in the United States use it according to the National Institutes of Health [7]. The World Health Organization has reported that 103 out of 129 countries use acupuncture [7]. As the use of acupuncture continues to grow worldwide, there is also a growing concern about adverse outcomes with the activity. Infections have been reported as the most common adverse event related to acupuncture [8]. This highlights the importance of further characterizing which infections occur and how to treat infections that may be related to acupuncture use. Here, we present a case of a patient who received acupuncture treatment, who subsequently had a very severe disseminated infection.

## Case presentation

A 54-year-old female patient with a past medical history of hypertension, bilateral glaucoma, and cataracts presented to the emergency department (ED) with recurrent fevers and encephalopathy in the setting of progressive right hip pain and left foot swelling over two weeks. Reportedly, the right hip pain began immediately after yoga class. She had increasing pain, causing reduced mobility, prompting a presentation to an urgent care for assessment five days later. At the time, an X-ray of her right hip showed only mild osteoarthritis; she was sent home with naproxen for pain management. The patient also pursued acupuncture and cupping to the right hip to help alleviate the pain, but had no improvement. She subsequently developed fevers, with her family noting drowsiness and intermittent confusion, and forgetfulness in the next few days after her acupuncture session, which prompted her to present to the ED.

Vitals in the ED showed a temperature of 102.4°F, heart rate of 92 beats per minute, respiratory rate of 20 breaths per minute, blood pressure of 133/56 mmHg, and oxygen saturation of 94% on 2 liters/minute via nasal cannula. The patient’s peak temperature was 102.9°F. Physical exam was notable for tachypnea, mild nonpitting edema, and erythema on the left dorsal foot and posterior bilateral extremities, mild diffuse erythema of the back, and a 1 cm scabbed lesion on the right gluteus. The patient otherwise had no other wounds or lesions noted on her skin.

Initial laboratory studies included a complete blood count remarkable for leukocytosis of 25,700 and a comprehensive metabolic panel remarkable for hyponatremia with sodium of 124 and potassium of 2.7, likely due to hypovolemia in the setting of decreased oral intake prior to presentation (Tables 1-2). Adrenal insufficiency was considered; however, the patient's vitals were hemodynamically stable. Urinalysis showed bacteria as well as hyaline casts and granular casts. Blood cultures and urine cultures were also obtained, but urine cultures were negative; thus, urinalysis was thought to have contamination. Computerized tomography (CT) brain with contrast was obtained to evaluate her altered mental status, which showed no abnormalities. To evaluate her lower extremity edema, X-ray of her left foot and left lower extremity Doppler ultrasound (US) were obtained, which were normal. CT abdomen and pelvis with contrast showed three 5 to 6 cm fluid collections in the right lower erector spinae, gluteus maximus, and iliopsoas muscles concerning for abscesses (Figures 1-2).

**Table 1:** Emergency room initial complete blood count and inflammatory lab results 10E3/uL: x 10^3 ^per microliter; g/dL: grams per deciliter; fL: femtoliter; mm/hr: millimeters per hour; ug/L: micrograms per liter

Blood work test	Test results	Reference range
White blood cell	25.70 x 10E3/uL	4.16-9.95 x 10E3/uL
Hemoglobin	12.8 g/dL	11.6-15.2 g/dL
Hematocrit	36.8 %	34.9-45.2
Mean cell volume	83.8 fL	79.3-98.6 fL
Platelet	102 x 10E3/uL	143-398 x 10E3/uL
Absolute neutrophil count	23.6 x 10E3/uL	1.8-6.9 x 10E3/uL
Erythrocyte sedimentation rate	88 mm/hr	<=25 mm/hr
Procalcitonin	2.87 ug/L	<0.010 ug/L

**Table 2:** Emergency room initial basic metabolic panel lab results mmol/L: millimole per liter; mg/dL: milligram per liter; mL/min/1.73 m2: milliliter per minute per 1.73 meters squared; mg/dL: milligram per deciliter; GRF: glomerular filtration rate

Blood work test	Test results	Reference range
Sodium	124 mmol/L	135-146 mmol/L
Potassium	2.7 mmol/L	3.6-5.3 mmol/L
Chloride	82 mmol/L	96-106 mmol/L
Total CO2	27 mmol/L	20-30 mmol/L
Anion gap	15 mmol/L	8-19 mmol/L
Urea nitrogen	29 mg/dL	7-22 mg/dL
Creatinine	0.90 mg/dL	0.60-1.30 mg/dL
Estimated GFR	76 mL/min/1.73m2	60-89 mL/min/1.73m2
Glucose	133 mg/dL	65-99 mg/dL

**Figure 1:**
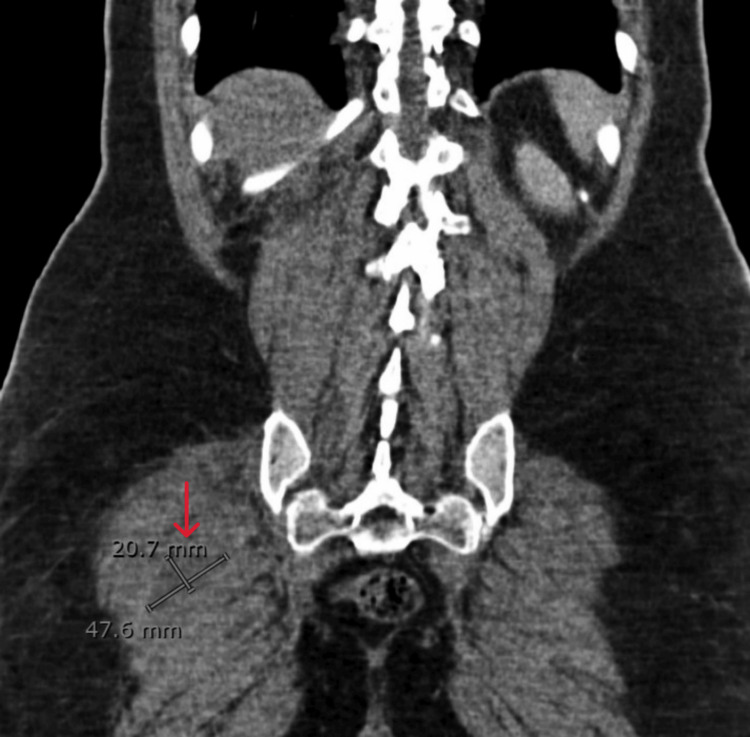
CT abdomen and pelvis with contrast showing one of the multiple abscesses in the patient’s right gluteus region CT: computerized tomography

**Figure 2:**
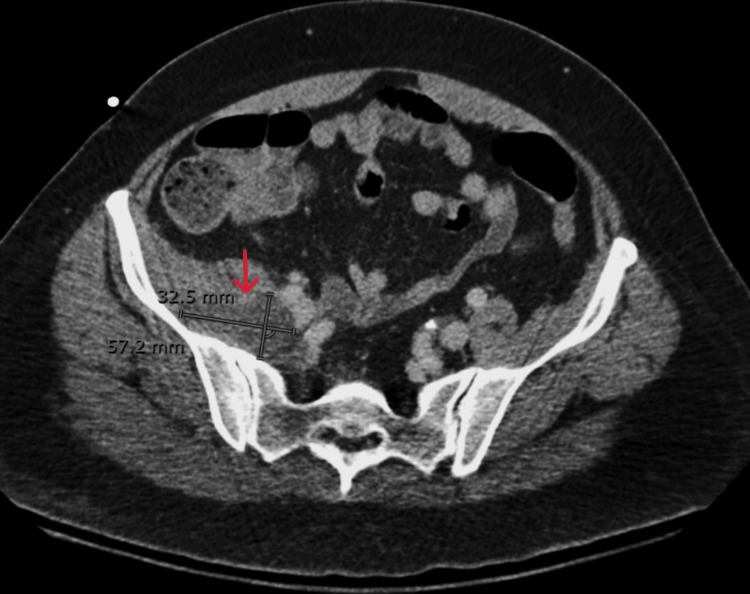
Additional views of CT abdomen and pelvis with contrast showing one of the multiple abscesses in the patient’s right gluteus region CT: computerized tomography

Initial treatment included sepsis bundle resuscitation with intravenous normal saline. She was started on empiric antibiotics consisting of ceftriaxone 2 g intravenous (IV) daily and vancomycin IV daily, dosed by pharmacy. Her transient hypoxia was likely due to pulmonary edema in the setting of sepsis versus atelectasis seen on chest X-ray. On hospital day 3, while the patient’s urine culture came back negative, her blood cultures, four out of four bottles, were growing MSSA. Infectious disease consultation was obtained. Antibiotics were changed from ceftriaxone and vancomycin to cefazolin 2 g IV every eight hours. An MRI of the lumbar spine and abdomen was obtained due to reported pain in these areas, and it revealed extension of the abscesses into the L5-S1 spinal canal and the right L4-L5 foramen with osteomyelitis (Figure 3).

**Figure 3:**
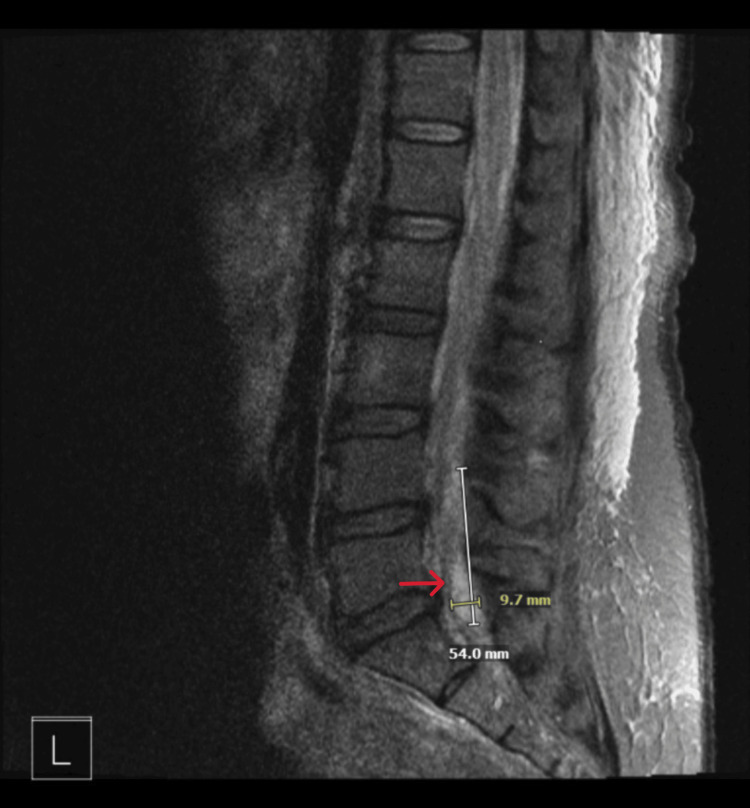
MRI lumbar spine showing one of the abscesses extending into the L5-S1 spinal canal MRI: magnetic resonance imaging

Given MSSA bacteremia, a transthoracic echocardiogram and a transesophageal echocardiogram (TEE) were obtained to definitively rule out endocarditis, which showed no valvular vegetations or abnormal structures, with a normal ejection fraction. Interventional radiology (IR) performed drainage of 10 milliliters from her right gluteal abscesses, which was cultured and grew MSSA. The orthopedic spine surgery performed an L4-L5 laminectomy on hospital day 5, given her infection of the spine. Daily blood cultures were continued to be drawn for the patient as they were persistently positive despite culture data showing sensitivity to cefazolin. Cefazolin dose was increased to 12 g IV every 24 hours per the infectious disease’s recommendation. Even with the dose increase, positive blood cultures persisted. On hospital day 11, the patient had worsening left foot edema; an ultrasound (US) was performed, showing a phlegmon. CT abdomen and pelvis was again performed to re-evaluate her abscesses, and it showed an increase in abscess size on the right gluteus from 5-6 cm to 10-11 cm. Repeat IR-guided aspiration of these abscesses was completed. Incision and drainage of the phlegmon of her left foot was performed by a podiatrist. Hospital day 12 was the first day on which the collected blood cultures were negative. The patient was ultimately discharged on hospital day 17 with a peripherally inserted central catheter (PICC) line for continued high-dose cefazolin for a total of six weeks. A summary table of the hospital course is depicted below (Table 3).

**Table 3:** Summary timeline of events and progression of data with treatments CT: computed tomography; IV: intravenous; MSSA: methicillin-sensitive *Staphylococcus aureus*; TTE: transthoracic echocardiography; TEE: transesophageal echocardiography; MRI: magnetic resonance imaging; IR: Interventional radiology; PICC: peripherally inserted central catheter

Hospital day	Summary of events
1-2	Workup showing leukocytosis and CT abdomen and pelvis showing three 5 to 6 cm abscesses in the right lower erector spinae, gluteus maximus, and iliopsoas muscles. Began initial empiric antibiotic treatment with ceftriaxone 2 grams IV daily and vancomycin IV daily, dosed by pharmacy, while awaiting further culture data from blood and urine cultures
3-4	Urine culture was negative, but blood cultures were growing MSSA. Increased ceftriaxone frequency from daily to every 8 hours for more aggressive treatment. TTE and TEE were obtained, which were negative for endocarditis. MRI lumbar spine showed paraspinal abscesses and osteomyelitis at L4-L5 and L5-S1. IR drainage of abscesses performed
5	L4-L5 laminectomy performed for source control
6-10	Daily blood cultures continued to be drawn and were persistently positive for MSSA. Cefazolin dose increased from 2 grams every 8 hours to 12 grams every 24 hours. Daily cultures remained positive despite the dose increase
11	The patient had worsening left foot edema with an ultrasound showing phlegmon. Repeat CT abdomen and pelvis showed an increase in right gluteus abscesses. IR drainage of gluteal abscesses and left foot phlegmon was performed
12	Blood cultures were negative
13-16	Continued to treat the patient with higher dose cefazolin at 12 grams every 24 hours
17	Patient was discharged with PICC to complete high-dose cefazolin at 12 grams every 24 hours for a total of 6 weeks

## Discussion

The use of acupuncture through the insertion of needles at specific body points has shown some evidence of improving pain, such as in our patient who sought acupuncture to relieve the hip pain she had after a yoga session. An examination of 16 review articles and 11 randomized control trials all suggest short-term pain relief benefits of acupuncture in those with symptomatic knee osteoarthritis and chronic low back pain [9]. There have been studies on the mechanism of how acupuncture might help alleviate symptoms. One such proposal is that the stimulation from needles could influence inflammatory markers, which in turn cause hormonal changes and immunomodulation [1]. Another theory is that analgesia occurs from the release of natural opioids [1]. Others have postulated that serotonin, norepinephrine, orexin, and endocannabinoid hormones are also released, leading to analgesia from acupuncture [10]. Dopamine may also be released from the nucleus accumbens, which could contribute to pain relief [10]. There are a few methods that acupuncturists use to sterilize needles, such as boiling or immersion in at least 75% medical grade alcohol [11]. Others disinfect needles through an autoclave with high pressure and high temperature [11]. There is no governing body that standardizes sterilization techniques and requirements for acupuncture. It is unclear whether proper sterilization techniques were used for our patient before she received acupuncture. 

In this case, we presented a patient who was found to have disseminated MSSA bacteremia that was likely due to receiving acupuncture, as she had no other risk factors or discernible inciting etiology, and did not have risk factors that would cause higher susceptibility to abscesses, such as diabetes or immunocompromise. There have been previous studies regarding adverse events seen in conjunction with acupuncture. No less than 239 reported cases of infections associated with acupuncture have been published [12]. Of these 239 cases, 193 were found to have *Mycobacterium *infection, and 19 had concerns of *Staphylococcus *infections, with nine of the cases attributed to methicillin-resistant *Staphylococcus aureus *infections and the rest being MSSA [12]. Other adverse events found in association with acupuncture include pneumothorax, central and peripheral nervous system injury, scarring, edema, aneurysm, and pseudoaneurysm development [12]. Thus, it is important that if one were to undergo acupuncture, it is handled by an experienced professional and proper sterilization techniques are utilized to decrease the risk of adverse events. If an infection is found in a patient with recent acupuncture, then providers should consider *Staphylococcus aureus *as a cause, as this serious infection may worsen without aggressive treatment.

Source control and aggressive treatment with early antibiotic use are important when treating sepsis and bacteremia, as in our patient who had multiple abscesses and phlegmon, showing the virulence of MSSA. Regarding antibiotic choice, there are different options used to treat MSSA. The European Society of Cardiology recommends flucloxacillin or cefazolin, while the American Heart Association recommends nafcillin or oxacillin [13]. There have been studies that have shown cefazolin had less incidence of nephrotoxicity and less mortality compared to other antistaphylococcal penicillins, and yet, it was noninferior to treatment of MSSA infections [13,14]. Cefazolin also has less frequent dosing compared to antistaphylococcal penicillins, which may be advantageous and is also an option in settings where patients have an allergy to penicillins [13,14]. Thus, using cefazolin compared to other antibiotics was preferred in our case for these reasons. But sepsis and MSSA bacteremia require more than antibiotics for treatment. Studies have shown that a third of patients have persistent bacteremia despite antibiotic therapy [15]. This was in the case of our patient who had not shown significant improvement after more than one week of antibiotic treatment. It was not until source control was obtained through a laminectomy, incision, and drainage of her abscesses and phlegmon, both by IR and podiatry, that she began to have symptomatic improvement and negative blood cultures. Without source control for infections such as our patient’s virulent MSSA, there was the risk of worsened outcomes, which have been seen in several studies. For example, a study called the European Society of Intensive Care Medicine-endorsed Abdominal Sepsis (ABSES) study found that those with intra-abdominal infections that did not achieve adequate source control within seven days had higher mortality risk and other complications [16]. With studies supporting the need for source control in sepsis, the 2021 Surviving Sepsis Campaign guidelines recommend source control as soon as possible as one of the best practices when treating sepsis [17].

## Conclusions

Having no other risk factors or possible source of MSSA bacteria, acupuncture is the most likely inciting cause of this patient’s infection; there was a strong temporal association, and there was no other trauma leading to loss of skin integrity. Acupuncture may be a useful modality for pain management; however, our case demonstrates the importance of proper sterilization techniques and expert care to lower the risk of infections through bacterial seeding. MSSA is a more common organism associated with acupuncture-related infection, as it is a common skin flora. MSSA’s pathogenicity and virulence are demonstrated by this patient’s widely disseminated infection, including abscesses in the gluteal muscles, spine, foot, and vertebral column. Early source control and proper antibiotic treatment are an essential part of treatment, as it was in this case.
